# The association between renal impairment and polypharmacy among older Palestinian patients: a multi-center cross-sectional study

**DOI:** 10.1186/s12875-023-02005-9

**Published:** 2023-02-16

**Authors:** Shayma Naghnaghia, Zaher Nazzal, Layan Abu Alya, Rowa’ AL-Ramahi, Zakaria Hamdan, Esra’a Samara

**Affiliations:** 1 Faculty of Medicine and Health Sciences, An-Najah National University, Nablus, P.O. Box 7, Palestine; 2 Department of Pharmacy, Faculty of Medicine & Health Sciences, An-Najah National University, Nablus, P.O.Box 7, Palestine; 3 Internal Medicine Department, An-Najah National University Hospital, Nablus, Palestine

**Keywords:** Elderly, Impaired renal function, Polypharmacy, Primary healthcare clinics

## Abstract

**Purpose:**

This study aimed to examine the association between renal impairment and polypharmacy among older Palestinian patients visiting primary healthcare centers and to examine potentially inappropriate medications among older patients.

**Methods:**

A cross-sectional study was conducted among PHC clinic attendees aged 65 and older. We used medical records and an interviewer-administered questionnaire for data collection. Participants with eGFR less than 60mls/min/1.73 m2 were categorized as renal impaired; we then calculated the prevalence of renal impairment and used Poisson multivariable regression model with robust variance to identify associated factors. Beer’s criteria and literature reviews were used to evaluate renal impairment patients’ medication and to determine the frequency of PIPs.

**Results:**

The study included 421 participants (224 female, 197 male), and 66.3% were between the ages of 65 and 75. The prevalence of renal impairment was 30.2% (95%CI: 25.8–34.6%). Polypharmacy [aPR = 2.7, 95%CI: 1.7–4.3], stroke [aPR = 2.6, 95%CI: 1.1–2.3], females [aPR = 1.7, 95%CI: 1.2–2.5], and older patients over the age of 80 [aPR = 2.4, 95%CI: 1.6–3.5] were the main factors associated with renal impairment. RAAS (54.3%), metformin (39.3%), and sulfonylurea (20.4%) were the most frequently reported PIP in renal impairment patients.

**Conclusion:**

This study demonstrates a relationship between polypharmacy and renal impairment. Some people with renal impairment receive drugs that those with kidney illness should avoid or use with caution. It is important to prescribe only necessary medication, choose non-nephrotoxic alternatives, and frequently monitor renal function.

## Introduction

Renal impairment is the end outcome of progressive and irreversible silent pathologic processes, which may progress to advanced stages without the patient’s complaints. Early detection of renal impairment helps patients avoid dialysis or a kidney transplant. According to the American Kidney Foundation, the estimated glomerular filtration rate used to classify renal impairment as a stage of chronic kidney disease (CKD) is greater than 90 mL/min with evidence of kidney damage in the first stage of CKD and less than 15 mL/min in the end stage of CKD [1, 2].

Renal impairment prevalence has increased significantly over the last few decades, indicating that renal impairment and its complications may burden the healthcare system [3–5]. Diabetes, hypertension, chronic glomerulonephritis, autoimmune diseases, polypharmacy, and the use of nephrotoxic medications are all significant risk factors [6, 7] and should be monitored closely if the kidney function of the patient is to be preserved. In addition, renal impairment increases patients’ cardiovascular risk and other comorbidities while negatively impacting their quality of life [8]. Few studies have evaluated the morbidity of CKD or renal impairment in Palestine. In 2013, the prevalence of end-stage renal disease patients on dialysis in Palestine was 240.3 per million of the population [9]. A study conducted in 2008, showed that 35.5% of patients with diabetes and high blood pressure had impaired renal function [10]. Another study found that nearly one-fourth of diabetic patients in Palestine had CKD [11].

For various reasons, the older population over 65 requires special attention from the health sector. Aging alters many physiologic processes, causing older people to have different definitions of diseases and different normal ranges of lab results. In addition, the glomerular filtration rate typically peaks in the third and fourth decades of life, then declines by about 8 mL/min/1.73 m^2^ per decade after [12]. All of these factors, including aging, comorbidities, hospitalization costs, and multiple prescription medications, contribute to the significance of the older population. Because primary healthcare (PHC) is the first line of prevention, it should be well-prepared with strategies to manage this age group [13], and one of the main challenges in dealing with this group of patients is polypharmacy, which is caused by their multiple comorbidities such as diabetes mellitus, hypertension, and cardiovascular disease.

Polypharmacy, commonly described as the use of more than five medications [14], is one of the challenges that has been increasingly apparent over the past few years and is something that both patients and physicians must deal with. Use of several medications is a manifestation of the high prevalence of comorbidities, each of which requires a different regimen to be managed well [15]. The most severe consequences of polypharmacy in older patients are an increased likelihood of inappropriate prescription usage and compliance and an increased risk of experiencing adverse drug effects [16].

Older people with renal impairment are at a high risk of using inappropriate prescriptions due to their multiple comorbidities, polypharmacy, and changes in the pharmacokinetics and pharmacodynamics of drugs excreted by the kidney due to a decreased filtration rate and renal metabolism [13]. Therefore, increasing awareness of polypharmacy and renal impairment in primary healthcare will aid in the prevention of many cases of inappropriate use and the development of clear strategies to guide physicians in the treatment of these patients and the management of their comorbidities in light of the renal impairment they already face [17].

Physicians should manage all medications properly when treating patients with renal impairment while keeping the renal effect in mind [1]. Especially medications that are nephrotoxic, whether they are inherently nephrotoxic or dose-or duration-dependent. A combination of more than one nephrotoxic drug increases the risk of renal impairment [18]. Previous studies have reported an association between polypharmacy and renal impairment, with the likelihood of renal impairment increasing significantly with the number of medications taken by older people [7, 19–21]. However, few studies have been conducted among PHC attendees, and no previous studies have investigated this problem among older Palestinians and the region. Therefore, the primary objective of this study is to examine the association between renal impairment and polypharmacy among older Palestinian patients visiting primary healthcare centers, and the secondary objective is to examine potentially inappropriate medications among older patients.

## Methodology

### Study design and population

An observational cross-sectional study was carried out in the three main Palestinian West Bank districts of Nablus, Jenin, and Tukaram between December 2021 and March 2022. The study was conducted at six PHC centers in each district. In Palestine, PHC centers are the first point of contact for the majority of the population, offering free preventive and curative services. Therefore, all patients older than 65 attending PHC clinics during the study period were considered eligible for the study. Those with communication difficulties, such as mental illnesses, hearing loss, and others, were excluded. A minimum sample size of 387 was calculated using the OpenEpi software, and the equation *n* = [DEFF*Np(1-p)]/ [(d2/Z21−/2*(N-1) + p*(1-p)], with an expected outcome of roughly 50%, precision 0.05, and a 95% confidence interval. We collected data from 421 eligible patients using a convenience sampling technique.

PHC clinic attendant patients over the age of 65 were initially asked if they agreed to participate voluntarily in the study after the nature and subject of the research were explained. Those who agreed to participate provided written informed consent and were interviewed in a clinic equipped with a high-privacy atmosphere. The interview did not take more than 7 min. Other information was completed from the patient’s file. We took all steps to protect the confidentiality of the participants’ collected data. Those with GFR < 60 were notified and advised to have additional follow-up measurements. The study protocol was approved by Institutional Review Board Al-Najah National University (Reference #: Farm. Med. Dec. 2021/2) and by the Palestinian Ministry of Health.

### Data collection and measures

An interviewer-administered questionnaire and a review of the patient’s medical files were employed to collect data. The questionnaire was developed based on a literature review [20, 22]. Its first part contains demographic information such as age, gender, weight, residency, marital and social status, monthly income, educational level, and living arrangement. The second section focused on the patient’s medical history and the list of medications used in the previous 2 weeks by type and name, prescription or over-the-counter, and where prescriptions were filled. The third section assessed medication side effects, hospitalization history over the previous 2 years, and average PHC visits, which according to Palestinian MOH, PHC chronic disease protocols should be every 3 months. The patient’s medical history was then evaluated to verify diagnoses and treatments.

Renal impairment was defined as an estimated glomerular filtration rate (eGFR) of <60mls/mins 1.73m^2^ [23]. The number of medications they took was used to evaluate polypharmacy, defined as taking five or more medications simultaneously [14]. The Beers criteria, a widely used tool that provides a list of medications whose risks outweigh their benefits in older people and should be avoided or used with caution, were used to identify potentially inappropriate medications (PIM) for each patient [24]. Potentially inappropriate prescriptions (PIP) are defined as medications that have inappropriate doses or are contraindicated according to the participant’s renal function [25]. To determine the PIMs, we reviewed each patient’s medications and evaluated them using Beer’s criteria and the literature on medications known to be inappropriate for use in patients with renal impairment [12, 26]. The questionnaire was piloted on 40 members of the research population after three experts in the field reviewed it. In addition, interviews were conducted by two authors, ES and SN, who are family physicians and have interviewing training.

### Data analysis plan

The IBM SPSS Statistics for Windows software, version 21 (IBM Corp., Armonk, NY, USA), was used to enter and analyze data. Continuous variables were presented as mean ± standard deviation (SD), and frequency and percentages were used to describe categorical variables. We computed the prevalence of renal impairment and its 95% confidence intervals (CIs). In univariate analysis, we used the Chi-square test to determine the association between renal impairment and polypharmacy. To identify factors associated with renal impairment, we utilized a multivariable Poisson regression model with robust variance, and the findings were reported as adjusted prevalence ratio (aPR) with a 95% Confidence Intervals (95%CI). We chose this model because odds ratios obtained in cross-sectional studies using logistic regression may overestimate prevalence ratio when the outcome is prevalent [27]. The model included variables found to be relevant in the literature and those determined to be significant by univariate analysis. All *P*-values were two-sided, and *P* < 0.05 was considered statistically significant.

## Results

### Descriptive characteristics

A total of 470 PHC patients were approached and invited to participate in the study; 421 agreed and completed it, yielding a response rate of 89.6%. More than half of the participants were female (53.2%), the majority (66.3%) were between the ages of 65 and 75, 89.1% lived with their families, and 66.3% were married. Regarding education, 59.1% had primary education or less, and 60.6% had a low income (Table 1). The majority (87.2%) visited PHC clinics more than once in the previous 3 months, and more than half (60.1%) admitted to getting their medication from more than one place. The most prevalent chronic disease based on medical history was hypertension (84.8%), followed by diabetes (56.0%) and heart disease (36.6%). One-fourth reported hospitalization within the preceding 2 years (Table 2).

**Table 1:** Participants’ background characteristics and their relationship to renal impairment (*n* = 421)

	Total	Renal Impairment		***P***-value*
	Frequency (%)	Yes (127) Frequency (%)	No (284) Frequency (%)	
**Gender**				
Male	197(46.8%)	40 (20.3%)	157 (79.7%)	<.001
Female	224(53.2%)	87 (38.8%)	137 (61.2%)	
**Age**				
65–69	144(34.2%)	29 (20.1%)	115 (79.9%)	<.001
70–74	135(32.1%)	37 (27.4%)	98 (72.6%)	
75–79	71(16.9%)	25 (35.2%)	46 (64.8%)	
≥ 80	71(16.9%)	36 (50.7%)	35 (49.3%)	
**Residency**				
Urban	228(54.2%)	61 (26.8%)	167 (73.2%)	.061
Rural	193(45.8%)	66 (34.2%)	127 (65.8%)	
**Educational level**				
Primary education or less	249 (59.1%)	93 (37.3%)	156 (62.7%)	<.001
High school	75 (17.8%)	17 (22.7%)	58 (77.3%)	
Diploma or higher	97 (23.1%)	17 (17.5%)	80 (82.5%)	
**Income per month** *(US Dollar)*				
Less than 600	255 (60.6%)	92 (36.1%)	163 (63.9%)	.003
600–1199	144 (34.2%)	32 (22.2%)	112 (77.8%)	
More than 1200	22 (5.2%)	3 (13.6%)	19 (86.4%)	
**Marital status**				
Married	279(66.3%)	74 (26.5%)	205 (73.5%)	.022
Unmarried	142(33.7%)	53 (37.3%)	89 (62.7%)

**Table 2:** The clinical characteristics of study participants and their relationship to renal impairment (*n* = 421)

	Total	Renal Impairment		***P***-value*
	Frequency (%)	Yes (127) Frequency (%)	No (284) Frequency (%)	
**eGFR** (*Mean* ± *SD*)	69 ± 19.5	46.3 ± 11.1 5	79.9 ± 12.3	
***PHC visits*** *(last three months)*				
one visit/ 3 months	54(12.8%)	17 (31.5%)	37 (68.5%)	.467
More than one visit /3 months	367(87.2)	110 (30.0%)	257 (70.0%)	
***Source of medication***				.013
One PHC clinic	168(39.9%)	40(23.8%)	128(76.2%)	
More than one PHC clinic	253(60.1%)	87(34.4%)	166(65.6%)	
***Diabetes Mellitus***				
Yes	237(56.0%)	75 (31.6%)	162 (68.4%)	.521
No	184(43.7%)	52 (28.3%)	132 (71.7%)	
***Heart diseases***				
Yes	154(36.6%)	46 (29.9%)	108 (70.1%)	.505
No	267(63.4%)	81 (30.3%)	186 (69.7%)	
***Hypertension***				
Yes	357 (84.8%)	117 (32.8%)	10 (15.6%)	.005
No	64 (15.2%)	10 (15.6%)	54 (84.4%)	
***Stroke***				
Yes	40 (9.5%)	18(45.0%)	22(55.0%)	.027
No	381(90.5%)	109(28.6%)	272(71.4%)	
***Polypharmacy***				
Less than 5*†*	105(24.9%)	19 (18.1%)	86 (81.9%)	<.001
5–9	281(66.7%)	88 (31.3%)	193 (68.7%)	
More than 10	35 (8.3%)	20 (51.4%)	15 (48.6%)	
**Hospitalization** (last two years)				
Yes	107(25.4%)	38 (35.5%)	225 (71.7%)	.163
No	314(74.6%)	89 (64.5%)	69 (64.5%)	

*eGFR* Estimated glomerular filtration rate, *PHC* Primary Health Care

### Renal impairment

The prevalence of renal impairment was 30.2% (95%CI: 25.8–34.6%). The average eGFR for the entire sample was 69 ± 19.5 mls/mins 1.73m^2^; for participants with renal impairment (eGFR <60mls/mins 1.73m^2^) was 46.3 ± 11.1 5 mls/mins 1.73m^2^ and 79.9 ± 12.3 for those with eGFR >60mls/mins 1.73m^2^. Univariate analysis revealed that renal impairment differed significantly by gender, age groups, marital status, educational level, income, source of prescriptions, and comorbidities, particularly hypertension, stroke, and polypharmacy (all *P*-values <.05) (Tables 1 and 2).

We used Poisson multivariable regression with robust variance to study factors independently associated with renal impairment (Table 3). There was an association between renal impairment and polypharmacy, with older patients taking more than ten medications 2.7 more likely to have renal impairment [aP-value = <.001, aPR = 2.7, 95%CI: 1.7–4.3], while those taking five to nine medications were 1.6 times more likely [aP-value = .028, aPR = 1.6, 95%CI: 1.1–2.4]. Other associated factors were found, too: females were 1.7 times more likely than males to have renal impairment [ap-value .002, aPR = 1.7, 95%CI: 1.2–2.5], and patients over the age of 80 were 2.4 times more likely to have renal impairment [aP-value = <.001, aPR = 2.4, 95%CI: 1.6–3.5], while those between the ages of 75 and 79 were 1.7 times more likely [aP-value = .020, aPR = 1.7, 95%CI: 1.1–2.6]. Older patients with stroke were 1.6 times more likely to have renal impairment than those without stroke [aP-value = .028 aPR = 1.6, 95%CI: 1.1–2.3].

**Table 3:** Multivariable Poisson regression (with robust variance) analysis of variables related to renal impairment

	Prevalence Ratio	95% CI	a*P*-value
**Gender** *(Ref: Male)*		1	
Female	1.7	1.2–2.5	.002
**Age** *(Ref: 65–69)*		1	
70–74	1.4	.93–2.1	.115
75–79	1.7	1.1–2.6	.020
≥ 80	2.4	1.6–3.5	< .001
**Residency** *(Ref: Urban)*		1	
Rural	1.2	.85–1.5	.418
**Educational level** *(Ref: Diploma or higher)*		1	
Primary education or less	1.1	.66–1.9	.696
High school	.96	.52–1.8	.885
**Income per month** *(Ref: More than 1200$)*		1	
Less than 600$	1.8	.61–5.1	.298
600$ -1200$	1.3	.46–3.9	.589
***Marital status*** *(Ref: Married)*		1	
Unmarried	1.1	.80–1.5	.599
***Hypertension*** *(Ref: No)*			
Yes	1.4	.80–2.5	.237
***Stroke*** *(Ref: No)*			
Yes	1.6	1.1–2.3	.028
***Polypharmacy*** *(Ref: Less than 5)*			
5–9	1.6	1.1–2.4	.028
More than 10	2.7	1.7–4.3	< .001

*Ref* Reference group, *aP-value* adjusted *P*-value, *CI* Confidence Interval

### Potentially inappropriate prescriptions in renal impairment patients

According to the updated Beers criteria, more than one-third (36.8%) of participants with renal impairment were found to have at least one PIM. The most frequent PIMs found among participants with renal impairment were sulfonylurea (20.4%), alpha-blockers (4.7%), PPI (4.7%), and NSAIDs (3.9%) (Table 4).

**Table 4:** The most frequent potentially inappropriate medications in renal impairment patients compared to those with normal renal function

	PIM in normal renal function patients (***n*** = 294)	PIM in renal impaired patients (***n*** = 127)
	Frequency (%)	Frequency (%)
**Long-acting sulfonylurea**	76 (25.7%)	26(20.4%)
**Alpha-blockers**	12(4.1%)	6(4.7%)
**Proton pump inhibitors**	6(2.0%)	6(4.7%)
**NSAID**	8(2.7%)	5(3.9%)
**Digoxin**	4(1.4%)	4(3.1%)
**Antihistamine**	3(1.0%)	3(2.4%)
**CNS medication**	7(2.4%)	2(1.6%)
**Anti-Parkinson**	3(1%)	1(0.8%)

On the other hand, the following are the PIP found among the study’s participants and were classified as follows: firstly, contraindicated medication in renal impairment, finding include: 3.9% were on metformin,1.5%on combination of ACEI and ARBs, and 0.7% on allopurinol. Secondly, medication to be used with caution, 54.3% were on Renin-angiotensin-aldosterone system (RAAS), 35.4% on an inappropriate dose of metformin,7.8% on spironolactone,3.9%on combination of both thiazide and loop diuretics, and 0.7% on a combination of both ACEI and spironolactone.

## Discussion

Renal impairment increases morbidity and mortality, and as it is a predictor of cardiovascular disease if detected early, it is preventable and treatable [28]. Unfortunately, renal impairment in older people is not well studied in Palestine and the region. An old survey of patients with hypertension and diabetes in hospitals found that 35.5% have renal impairment [10]. Our study found that the prevalence of renal impairment among Palestinian older people is 30%, which is among the highest when compared to the global prevalence: 40% in the USA [4], 25.7% in Italy [29], 21.4% in Brazil [5], and 11.4% in China [30]. Healthcare providers and policymakers should pay more attention to this to adopt strategies to aid in the early detection of renal impairment to prevent and control it in its early stages, thereby reducing healthcare costs.

It’s known that the risk factors of renal impairment are complicated and multifactorial. Consistent with previous studies, Poisson multivariate regression reveals that, besides polypharmacy, female gender, increased age, and the presence of a stroke are significantly associated with impaired renal function. Polypharmacy, identified as a significant risk factor for potentially inappropriate medication [31], has also been linked to renal impairment in the older; the likelihood of renal impairment increases as the number of prescribed medications increases. Our results showed that older people with excessive polypharmacy (more than ten medications) are 2.7 times more likely to have renal impairment than those with less than five medications. This is consistent with the literature, which shows that polypharmacy exposure is significantly associated with an increased risk of kidney dysfunction [7, 19, 21]. Knowing that pharmacokinetic changes happen with aging, where renal elimination decreases, increase the risk of pharmaceutical ingredient and metabolite accumulation, which increases the risk of renal impairment [12]. Polypharmacy raises the risk of adverse drug events, drug-drug interactions, and drug-disease interactions, which increase the risk of renal impairment [20]. These findings highlight the importance of implementing strategies to reduce polypharmacy risk factors in the elderly, such as having a single source for medication prescriptions, managing frequent PHC clinic visits, and reducing unnecessary prescriptions by physicians.

Females were 1.7 times more likely to have renal impairment than males, which is consistent with previous literature [6, 7, 22]. This could be due to the differences in pathophysiology between males and females. Increased age significantly affects renal impairment, particularly in the above 70 people. Patients over 80 years old were found to be 2.4 times more likely to have renal impairment compared to younger age groups. Increasing age is responsible for some of the pathophysiology of renal impairment, where degeneration of sodium content, endothelial function, and renin-angiotensin system happens [29]. This could be due to multiple chronic diseases, especially hypertension and diabetes mellitus associated with aging, leading to decreased renal function. This finding should raise the awareness of PHC physicians treating those age groups about the risks of renal impairment, the importance of closely monitoring their kidney function to detect and treat any impairment early, and the importance of controlling chronic diseases that affect renal function.

Speaking of chronic diseases, stroke showed a significant association with renal impairment; older patients with stroke were 1.6 times more likely to have renal impairment. It is important to note that this is a two-way relationship: while renal impairment is thought to be a predictor of poor clinical outcomes and mortality after stroke, stroke has been found to increase the risk of renal impairment [32, 33]. This could be because stroke patients have poorer general health and less controlled chronic diseases and thus are predisposed to develop renal impairment [34]. This brings us back to the importance of early detection, treatment, and close monitoring of chronic diseases in the elderly, particularly those known to impair renal function, such as hypertension and diabetes. Patients who have had a stroke require close monitoring of their renal function.

More than one-third of study participants with renal impairment had at least one PIM, with long-acting sulfonylurea being the most common (20.4%). Sulfonylureas, in general, may cause prolonged hypoglycemia, which can be fatal in older people [24]. In addition, in patients with renal impairment, this medication’s side effects increased as their renal clearance decreased, resulting in more hypoglycemic episodes and potentially fatal consequences [26]. On the other hand, it was found that some patients with renal impairment used nonsteroidal anti-inflammatory medications, even though NSAIDs are known for their nephrotoxic effect by reducing renal flow, causing prerenal failure and acute tubulointerstitial nephritis; this should raise physicians’ awareness to look for a non-nephrotoxic alternative for pain management in this group [12].

Metformin is known to induce lactic acidosis, especially in patients with impaired renal function. The recommendation is to closely monitor renal function and adjust the dose or discontinue it accordingly [12, 26]. However, 3.9% of study participants with renal impairment were on metformin while their GFR was less than 30, and 35.4% were in inappropriate doses according to their GFR level. This emphasizes the importance of assessing renal function before starting metformin, closely monitoring it, and adjusting the dose accordingly. In addition, some of the study’s participants were on a combination of ACEI and ARBs, while others were on medications to be used with caution, including medications that need close monitoring to avoid renal function deterioration and electrolyte disturbance and need a dose adjustment. More than half of the study’s participants with renal impairment were on RAAS, less than 10% were on spironolactone, and a minority were on a combination of ACEI and spironolactone; those mentioned medications need close monitoring of serum potassium and renal function, as they may lead to hyperkalemia in renal impairment patients, which may be lethal by causing cardiac arrhythmias. Moreover, 4% were on a combination of both loop diuretics with thiazide, which is known to increase the risk of both hyponatremia and hypokalemia, wherefore need close monitoring of serum electrolytes [35, 36].

### Study strengths and limitations

To our knowledge, this is one of the few studies to report the association between polypharmacy and renal impairment among PHC patients and the first one to study nephrotoxic drugs in renal impairment patients in Palestine. However, the study’s findings should be interpreted with some limitations. First, due to the study’s cross-sectional design, data were collected at a single point in time, which may incur bias. The second was that psychiatric medications could not be assessed, despite many being considered PIM in patients with renal impairment. Finally, we could not determine the chronicity of renal disease due to the lack of two creatinine readings separated by 90 days. Instead, we used renal impairment to describe the decline in renal function.

## Conclusion

This study shows that polypharmacy was associated with renal impairment. Furthermore, stroke, gender, and age were found to be other important associated factors. Some patients with renal impairment receive medications that should be avoided or used with caution in those with kidney diseases. PHC physicians should cautiously prescribe medications to these patients. Further studies, especially prospective cohort studies, is required to ascertain whether medication treatment, as opposed to comorbid diseases, is responsible for renal impairment.

## Data Availability

All data generated or analyzed during this study are included in this published article.
